# Problems with mapping the auroral oval and magnetospheric substorms

**DOI:** 10.1186/s40623-015-0336-6

**Published:** 2015-10-12

**Authors:** E. E. Antonova, V. G. Vorobjev, I. P. Kirpichev, O. I. Yagodkina, M. V. Stepanova

**Affiliations:** 1 Skobeltsyn Institute of Nuclear Physics, Lomonosov Moscow State University, Moscow, Russia; 2 Polar Geophysical Institute, Apatity, Murmansk Region Russia; 3 Space Research Institute RAS, Moscow, Russia; 4 Physics Department, Science Faculty, Universidad de Santiago de Chile, Santiago, Chile

## Abstract

Accurate mapping of the auroral oval into the equatorial plane is critical for the analysis of aurora and substorm dynamics. Comparison of ion pressure values measured at low altitudes by Defense Meteorological Satellite Program (DMSP) satellites during their crossings of the auroral oval, with plasma pressure values obtained at the equatorial plane from Time History of Events and Macroscale Interactions during Substorms (THEMIS) satellite measurements, indicates that the main part of the auroral oval maps into the equatorial plane at distances between 6 and 12 Earth radii. On the nightside, this region is generally considered to be a part of the plasma sheet. However, our studies suggest that this region could form part of the plasma ring surrounding the Earth. We discuss the possibility of using the results found here to explain the ring-like shape of the auroral oval, the location of the injection boundary inside the magnetosphere near the geostationary orbit, presence of quiet auroral arcs in the auroral oval despite the constantly high level of turbulence observed in the plasma sheet, and some features of the onset of substorm expansion.

## Findings

### Introduction

Accurate mapping of the auroral oval onto the equatorial plane is necessary in determining the locations of substorm expansion phase onsets. In very early studies of auroral morphology, Akasofu (1964) showed that substorm onset is characterized by the brightening of the most equatorward auroral arc. Subsequent studies of dispersionless substorm injections (see Lopez et al. 1990 and Spanswick et al. 2010 and references therein) demonstrated that they are located near geostationary orbit, indicating that such injections take place deeply inside the magnetosphere. During the last few decades, however, the majority of studies of auroral substorms have focused predominantly on the geomagnetic tail and its dynamics. These studies have often relied on mapping the auroral oval structures to the equatorial plane along the magnetic field lines, calculated using various geomagnetic field models. This overall mapping approach has been termed “topological mapping” by Paschmann et al. (2002). However, the use of differing geomagnetic field models in the mapping process has produced conflicting results (see, for example, Hones et al. 1996, Weiss et al. 1997, Antonova et al. 2006, Xing et al. 2009).

Exclusion of a number of current systems from the geomagnetic field models serves to constrain the accurate topological mapping of auroral structures. For instance, existing models with a fixed predefined current geometry do not incorporate the current systems introduced by Antonova and Ganushkina (2000) and Antonova (2000), or the cut ring current (CRC). The topology of the CRC is very similar to the topology of the ordinary ring current (RC), and as such it can be considered a high latitude continuation of the ordinary ring current. The CRC occurs due to the fact that gradients of plasma pressure in the equatorial plane are predominantly directed towards the Earth at all magnetic local times (MLT), as shown by data from the Active Magnetospheric Particle Tracer Explorers/Charge Composition Explorer (AMPTE/CCE) satellite obtained at geocentric distances up to 8.8 R_E_ (Lui and Hamilton 1992; DeMichelis et al. 1999) and later results from the Time History of Events and Macroscale Interactions during Substorms (THEMIS) mission (Kirpichev and Antonova 2011; Wang et al. 2011; Antonova et al. 2013) at greater distances, up to the dayside magnetopause. This suggests that the current system surrounding the Earth extends almost up to the dayside magnetopause on the dayside and is located between around 6–7 and 10–12 R_E_ near midnight. However, the characteristics of the geomagnetic field differ between the nightside and the dayside regions. On the nightside, the surface of minimum magnetic field values is located at the equatorial plane. Therefore the nighttime transverse currents are also concentrated near the equatorial plane. In contrast, on the dayside, due to the compression of the magnetic field lines, there are two surfaces of minimal magnetic field values shifted away from the equatorial plane, with one in each hemisphere. This leads to the dayside transverse current being spread along a field line, causing the integral current to be split into two branches, which leads to the appearance of CRC-type current lines. This type of current lines also appears in the magnetohydrodynamic (MHD) models of high latitude transverse currents (Liemohn et al. 2011). Initial estimates of CRC intensities have been made by Antonova et al. (2009a, b) and Kirpichev and Antonova (2014). Thus, the existence of the CRC, which is not included in existing geomagnetic field models with fixed geometry, poses a problem for mapping the auroral regions to the equatorial plane, or, more exactly, to the surface of minimal magnetic field values.

Since the very beginning of space exploration, direct measurements have shown (see Paschmann et al. 2002 and references therein) that the region between the plasmapause and daytime magnetopause is filled with plasma similar to that in the plasma sheet. Additionally, plasma sheet-like precipitations have been observed in the auroral oval at all MLTs, including the near noon region (Newell and Meng 1992; Starkov et al. 2002). Therefore, it is natural to assume that the auroral oval may be mapped onto the ring-shaped structure surrounding the Earth, which is filled with plasma similar to that in the near-Earth plasma sheet. To verify this important assumption, it is necessary to develop a mapping technique that is based on specific plasma features, so-called “natural tracers”, which remain invariant along magnetic field lines. Such mapping has been termed “morphological mapping” (see Chapter 5 of the review by Paschmann et al. 2002).

Plasma pressure can be considered one such natural tracer, considering that the isotropic plasma pressure is conserved along a magnetic field line in the absence of field-aligned potential drop (Goertz and Baumjohann 1991) when the plasma is in magnetostatic equilibrium. Kirpichev and Antonova (2011) and Antonova et al. (2013) obtained the averaged distributions of plasma pressure, pressure anisotropy, and magnetic field near the equatorial plane at geocentric distances of >6 R_E_ for all magnetic local times (MLT), using data from the THEMIS mission. Later, Antonova et al. (2014a) calculated the values of plasma parameter “beta”, which represents the ratio of plasma pressure to magnetic pressure, in the region of minimal magnetic field values for all MLTs, using the Tsyganenko-2001 geomagnetic field model. They showed the existence of a high-beta plasma ring surrounding the Earth. The values obtained for the currents generated by corresponding plasma pressure gradients are sufficiently strong to produce significant distortions of the geomagnetic field.

In this study, we analyze the problem of auroral oval mapping without geomagnetic field models. Our mapping is based on a comparison between plasma pressure distributions at ionospheric altitudes and those in the equatorial plane. The feasibility of such an analysis in regions with very low geomagnetic activity has been previously demonstrated by Antonova et al. (2014b).

#### Methodology of morphological mapping

We compare the ion pressure distributions between low altitudes and the equatorial plane, assuming that plasma is in magnetostatic equilibrium:

1 $$ \left[\mathbf{j}\times \mathbf{B}\right]=\nabla P, $$

where **j** is the current density, **B** is the magnetic field, and *P* is the plasma pressure. This condition describes the plasma configuration when the plasma velocity is much smaller than Alfvén and sound speeds. Previous studies have shown that plasma pressure is nearly isotropic in the high latitude nightside magnetosphere (Kirpichev and Antonova 2011; Wang et al. 2011, 2012; Antonova et al. 2013, 2014a), and on the dayside, the pressure anisotropy is less than 1.2–1.4. In magnetostatic equilibrium, the isotropic plasma pressure is nearly constant along magnetic field line and can be considered a “natural tracer” or a landmark of the field line when the plasma anisotropy is low (see, for example, Dubyagin et al. 2002, 2003). The auroral acceleration also affects the condition in which *P* = const along a field line, decreasing the ion pressure in the regions of upward field-aligned currents. In contrast, in the regions of downward field-aligned currents, the pressure is generally conserved (see the review of Paschmann et al. 2002). This means that the ion pressure measured at altitudes lower than the auroral acceleration region will be equal to or less than the ion pressure in the equatorial plane.

The distribution of plasma pressure at auroral latitudes has been obtained by Wing and Newell (1998) and Wing et al. (2013), using data from Defense Meteorological Satellite Program (DMSP) satellites. Wing and Newell (1998) used the modified Tsyganenko-1989 model to map the pressures from auroral latitudes into the equatorial plane. The plasma pressures in the plasma sheet at geocentric distances >10 R_E_ are shown in Plate 1 of Wing and Newell (1998) and are larger than 0.2 nPa. It is well known that the typical value of the Bx component of the magnetic field in the tail lobes is around 20 nT, which gives a corresponding pressure of 0.16 nPa. Considering the total pressure balance across the plasma sheet, the plasma pressure in the center of the sheet should have a similar value (see Petrukovich et al. 1999 and references therein). Wang et al. (2001) compared the radial profiles of plasma pressure near to midnight (see Fig. 4 in this paper), obtained using the data from several high-apogee satellites and by mapping the DMSP ion pressure from ionospheric altitudes onto the equatorial plane (see Figure 4 of Wang et al. 2001). They showed that the plasma pressure obtained by Wing and Newell (1998) by mapping DMSP ion pressure onto the equatorial plane from ionospheric altitudes is larger than that measured by Explorer 45, International Sun-Earth Explorer (ISEE) 1 and 2, and Active Magnetospheric Particle Tracer Explorers/Ion Release Module (AMPTE/IRM) satellites on the equatorial plane. Tsyganenko and Mukai (2003) show that the averaged value of ion pressure obtained in the plasma sheet using Geotail data is equal to 0.229 nPa. Comparison of this value of plasma pressure with the values of ion pressures shown in Plate 1 of Wing and Newell (1998) also indicate that under quiet geomagnetic conditions the ion pressure obtained using the DMSP data is larger than that obtained in the plasma sheet using Geotail data. Therefore, under quiet geomagnetic conditions, the auroral oval must be mapped closer to the Earth than is possible using the Tsyganenko-1989 model. Recently, Wing et al. (2013) obtained the distribution of plasma pressure at the latitudes of the auroral oval for different phases of a magnetospheric substorm, using DMSP satellite data (see Plate 6 of Wing et al. 2013). In this, plasma pressures were found to range predominantly from 0.2 to 0.8 nPa. However, Wing et al. (2013) did not investigate the possible relationships between pressure distributions and different types of auroral electron precipitation.

Vorobjev and Yagodkina (2005) and Vorobjev et al. (2013) developed the Interactive Auroral Precipitation Model (APM) (PGIA 2015) based on the classification of electron precipitation types proposed by Starkov et al. (2002). In this model, the auroral oval precipitation (AOP) region is the area of structured precipitation within the auroral oval, the diffuse auroral zone (DAZ) is the region of diffuse auroral precipitation located equatorward of the AOP, and the soft diffuse precipitation (SDP) area is the region of soft diffuse precipitation located poleward of the AOP. Electron precipitating fluxes were measured by the DMSP F6 and F7 satellites in 1986 (APL Space Department 2015), which was a year of minimum of solar activity. All data were divided into eight MLT sectors, and the level of geomagnetic activity was characterized by AL and Dst indices. Inside each sector, the relationships between the corrected geomagnetic latitudes of the boundaries of each type of precipitation and the geomagnetic activity were established using a generalized regression technique. The results of this model provide distributions of the different types of auroral electron precipitation, as a function of corrected geomagnetic latitude and local geomagnetic time under different levels of geomagnetic activity.

Ion pressure is the dominant contributor to plasma pressure and can be obtained through analysis of the auroral ion precipitating fluxes, as previously shown by Wing and Newell (1998), Stepanova et al. (2004, 2008), and Wing et al. (2013). Comparison of ion pressure distributions, obtained in this way, with the boundaries of AOP, DAZ, and SDP enables the determination of ion pressures for each region. A comparison of these values with the pressure in the equatorial plane can thus provide important information about the mapping of the auroral oval.

#### Results of morphological mapping of the AOP and discussion

**Fig. 1:**
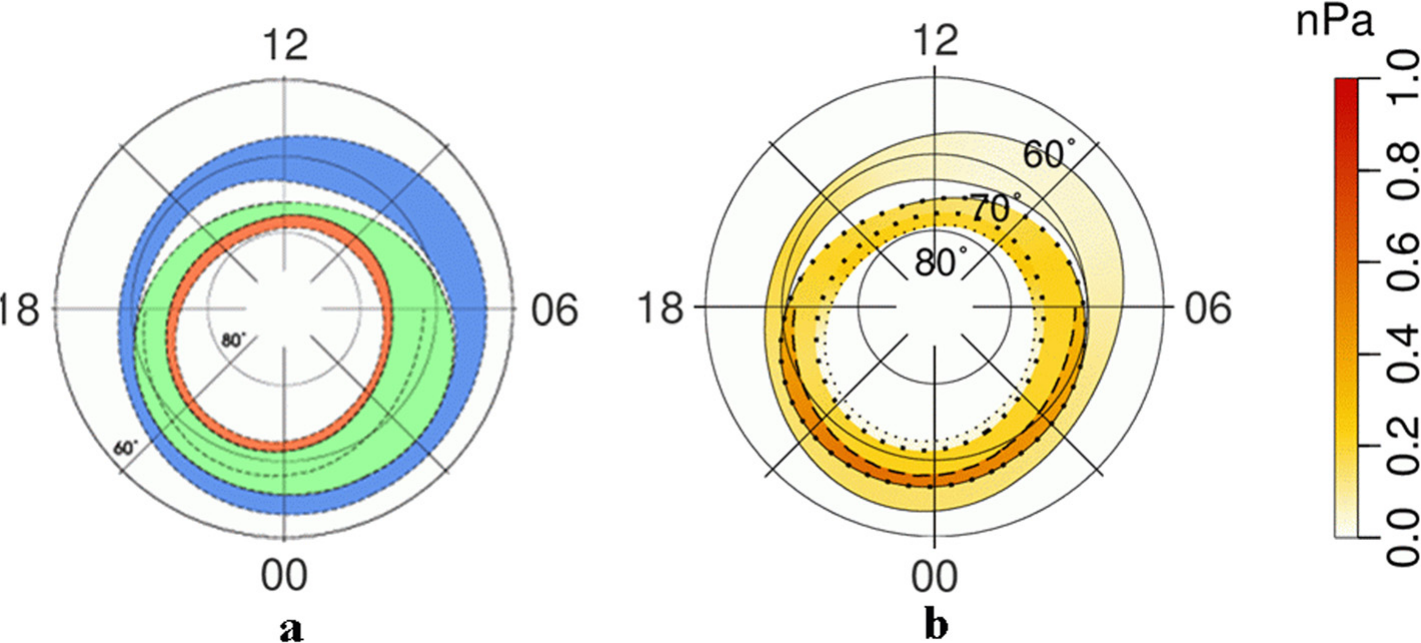
Location of **a** the AOP, DAZ, and SDP regions, and **b** the global distribution of ion pressures during quiet conditions. **a** Location of the AOP (*green*), DAZ (*blue*), and SDP (*red*) regions, and **b** the global distribution of ion pressure in the range 0.3–30 keV at ionospheric altitudes of around 110 km, obtained using data from the DMSP satellites for very quiet geomagnetic conditions of AL = −100 nT and Dst = −5 nT

**Fig. 2:**
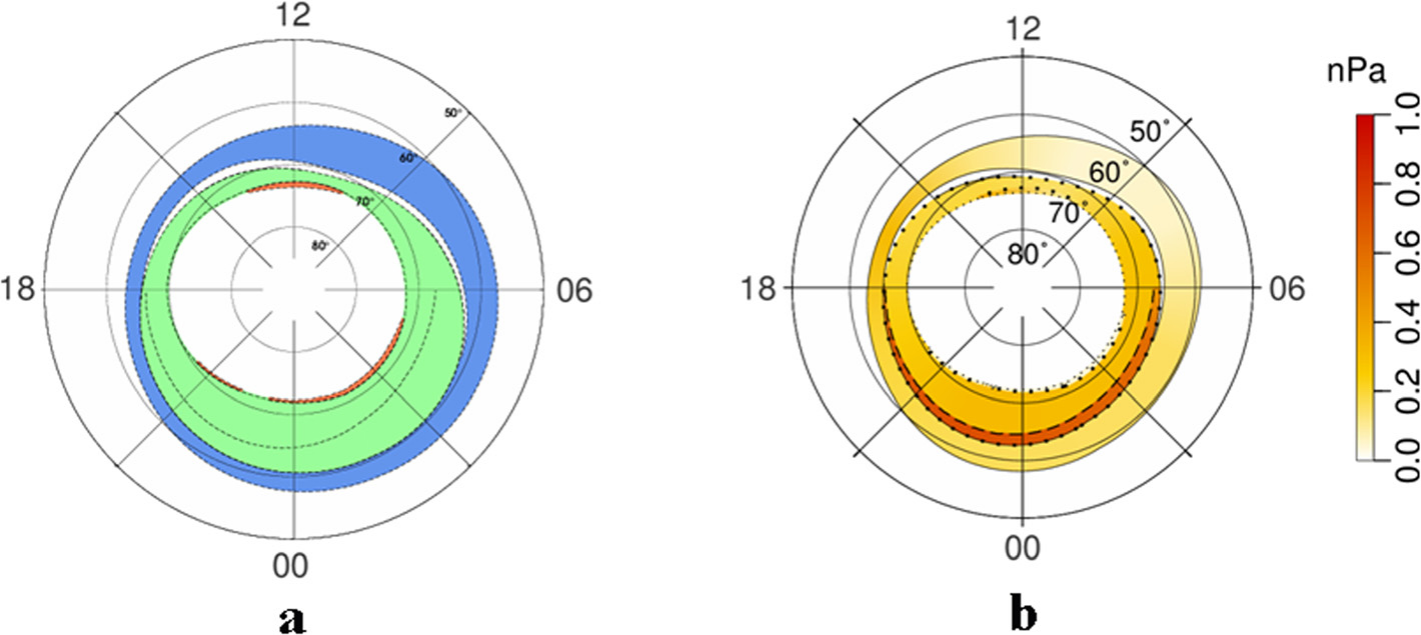
Location of **a** the AOP, DAZ, and SDP regions and **b** the global distribution of ion pressures during disturbed conditions, as for Fig. 1, but for moderately disturbed geomagnetic conditions of AL = −600 nT and Dst = −20 nT

Figure 1a shows the location of the AOP (green), DAZ (blue), and SDP (red) regions under very quiet geomagnetic conditions (AL = −100 nT, Dst = −5 nT). As can be seen from this figure, all three regions have a ring-like shape. Figure 1b shows the global distribution of ion pressures obtained from DMSP data for the same time interval and under the same geomagnetic conditions. The pressures are indicated by the color scale on the right-hand side of the figure. Bold dotted lines indicate the locations of the polar and equatorial boundaries of the AOP region, defining the traditional auroral oval; while the thin dotted line indicates the polar boundary of the SDP area. Figure 2a, b shows the locations of the AOP, DAZ, and SDP regions, as well as plasma pressures and region boundaries under moderately disturbed geomagnetic conditions (AL = −600 nT, Dst = −20 nT). As expected, the area covered by the auroral oval increases with the increase in geomagnetic activity. Figures 1b and 2b indicate that over most of the AOP region, the ion pressure is larger than 0.2 nPa under both quiet and disturbed conditions. It can also be seen that the ion pressure reaches around 1 nPa at the equatorial boundary of the AOP region. The pressures in the SDP are typically lower than 0.2 nPa. It should be noted that the ion pressure is smaller than the total plasma pressure, as the electron pressure is not considered, and therefore the corresponding pressure in the equatorial plane should be even higher.

**Fig. 3:**
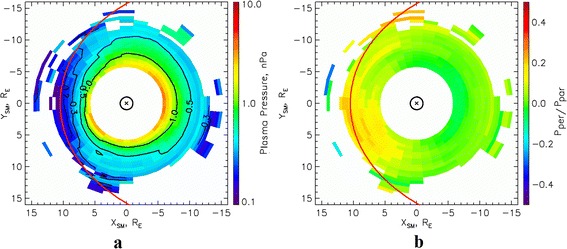
Plasma pressure and pressure anisotropy in the equatorial plane during low geomagnetic activity. Results of the calculation of **a** pressure and **b** pressure anisotropy in the equatorial plane, from data of the THEMIS mission, for AL = −100 nT and Dst = −5 nT

**Fig. 4:**
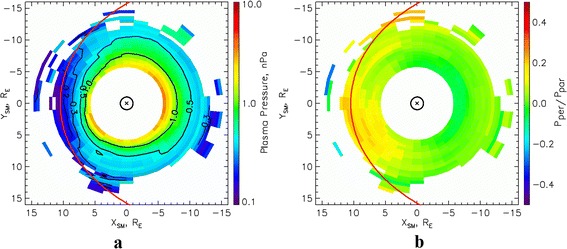
Plasma pressure and pressure anisotropy in the equatorial plane during high geomagnetic activity. Results of the calculation of **a** pressure and **b** pressure anisotropy in the equatorial plane, from data of the THEMIS mission, for AL = −600 nT and Dst = −20 nT

Figures 3a, b and 4a, b show the distributions of pressure at the equatorial plane and the pressure anisotropy obtained from THEMIS measurements, averaged within the same MLT intervals under the same geomagnetic conditions as those in Figs. 1 and 2, respectively. The black lines in Figs. 3a and 4a show the contours of constant pressure in the equatorial plane. Red lines show the positions of the magnetopause, obtained using the model presented by Shue et al. (1998). It can be seen from these figures that the pressure increases with an increase in geomagnetic activity. For instance, at the boundary of the plasma ring surrounding the Earth, the pressure is around 0.2 nPa during the quiescent period, but increases to around 0.3 nPa during moderately disturbed conditions. Analysis of the levels of anisotropy (Figs. 3b and 4b) indicates that anisotropy decreases with an increase in geomagnetic activity on the dayside. It should be noted that the dataset used to construct Fig. 3 is much larger than that for Fig. 4. The distributions obtained in this way are in good agreement with the nighttime pressure distributions at geocentric distances of more than 10 R_E_ provided by the statistical model of Tsyganenko and Mukai (2003).

Comparing Figs. 1b and 3a, it can be seen that the ion pressures across the majority of the AOP region are in good agreement with the total (electron and ion) pressures in the equatorial plane in the plasma ring surrounding the Earth. The same conclusion can be reached through an analysis of Figs. 2b and 4a. Differences of around 15 % of the total values are observed in the region of the downward field-aligned currents and can be much greater in the upward field-aligned current regions. However, in both cases, the total plasma pressure at the equatorial plane is larger than the ion pressure at ionospheric altitudes. Large values of ion pressure in the most part of AOP region mean in such a case that AOP is not mapped to the plasma sheet proper, for which the plasma pressure is smaller than 0.2 nPa. It is mapped into the surrounding of the Earth plasma ring. However, more exact AOP mapping requires more careful analysis, including the calculations of field-aligned potential drop and its effect on the ion precipitations leading to reduction of the ion pressure. Plasma sheet proper can be mapped into SDP where DMSP plasma pressure does not exceed 0.2 nPa.

Obtained AOP mapping without using any magnetic field model with predefined geometry of current systems can help to explain some features of auroral and substorm dynamics. For instance, mapping of the main part of the AOP region into the plasma ring surrounding the Earth can logically explain the well-defined circular shape of the auroral oval.

The existence of a drop in field-aligned potential is a very important feature of auroral dynamics. Simple evaluations have shown (see, for example, Kornilov et al. 2008) that in the absence of such a field-aligned potential drop, the energy flux of precipitating electrons is near to the registration threshold of polar imagers (around 0.5 erg cm^−2^ s^−1^) and ground-based measurements. This implies that only upward field-aligned currents can produce visible auroras, which is supported by the comprehensive analyses of Ohtani et al. (2010) and Korth et al. (2014). The location of the upward region 2 current sources (Iijima and Potemra 1976) inside the magnetosphere at comparatively small geocentric distances is well known. Further, analysis of the results of Xing et al. (2009) demonstrates that the source of upward-aligned region 1 currents is at a geocentric distance of around 11 R_E_, which corresponds to a location inside the plasma ring surrounding the Earth.

Over a long period of scientific investigation, the existence of a dispersionless substorm injection boundary near to the geostationary orbits has been difficult to understand (see Lopez et al. 1990; Spanswick et al. 2010 and references therein), under the assumption that the region of substorm onset is mapped into the geomagnetic tail. Isolated substorm onset was identified by Akasofu (1964) at the equatorial boundary of the auroral oval, without any auroral activity to the north (see the reviews of Akasofu 2004). According to our results, the AOP region equatorial boundary is mapped to a geocentric distance of 6–7 R_E_, which supports the models of substorm onsets occurring near to Earth, such as current disruption (see the review of Lui 2011) and magnetosphere–ionosphere interactions (Antonova 2002; Stepanova et al. 2002).

One of the main problems in understanding the high latitude magnetosphere and substorm dynamics is the high-level turbulence that is constantly observed within the plasma sheet. Intensive studies of this turbulence began with the identification and analysis of bursty-bulk-flow (BBF) events by Baumjohann et al. (1989, 1990) and continued with the investigation of different aspects of the turbulence, including eddy-diffusion, scaling, and intermittency (see, for example, Borovsky et al. 1997; Angelopoulos et al. 1999; Antonova et al 2000; Troshichev et al. 2002; Vörös et al. 2004; Uritsky et al. 2009). However, it is also well known that auroral arcs are well defined and can be very stable between geomagnetic disturbances. For this reason, it is necessary to explain the co-existence of stable auroral arcs in the auroral oval with the BBFs and associated turbulent fluctuation in the plasma sheet. The mapping of the AOP region to the plasma ring surrounding the Earth removes this problem, thanks to the braking of high-speed ion flows in the near-Earth central plasma sheet at geocentric distances of more than 9 R_E_ (Shiokawa et al. 1997), as well as a decrease in the degree of plasma sheet fluctuation at geocentric distances less than around 10 R_E_ (Stepanova et al. 2009, 2011; Pinto et al. 2011).

### Conclusions

Our analysis demonstrates the necessity to improve existing mapping techniques by using specific plasma features, so-called “natural tracers”, which conserve their characteristic signatures along magnetic field lines. We show here that plasma pressure can be successfully used as one such “natural tracer”.

A comparison of the pressure distributions measured in the equatorial plane by the THEMIS satellites with those measured by DMSP satellite above the auroral oval under both quiet and moderately disturbed geomagnetic conditions, reveals the role of the plasma ring surrounding the Earth in substorm dynamics. The results indicate that the popular approach of mapping the auroral oval into the plasma sheet must be modified and that the main part of the oval, especially its equatorial boundary, should not be mapped into the plasma sheet proper, but rather into the plasma ring surrounding the Earth. Such mapping explains the ring-like shape of the auroral oval, the location of the injection boundary inside the magnetosphere near the geostationary orbit, and the presence of quiet auroral arcs in the auroral oval despite the high level of plasma turbulence constantly observed in the plasma sheet. Subsequent studies to further verify and enhance the precision of these results should focus on an analysis of the processes occurring at geocentric distances between around 6–7 and 10–12 R_E_.

## Abbreviations

AOP: auroral oval precipitation
APM: auroral precipitation model
CRC: cut ring current (a high latitude continuation of the ordinary ring current)
DAZ: region of diffuse auroral precipitation located equatorward of the AOP
RC: ordinary ring current
SDP: region of soft diffuse precipitation located poleward of the AOP
